# Exploration of factors influencing the insufficient rate of tetanus vaccination among young women in an urban health zone: A cross-sectional study

**DOI:** 10.1097/MD.0000000000046605

**Published:** 2026-01-02

**Authors:** Eric Buchangende Ndagano, Rodrigue Fikiri Bavurhe, Zehra Sadia, Rabbia Munsab, Calvin R. Wei, Christian Bisimwa Wabene, Prudent Mituga Ngabe, Aymar Akilimali, Jonathan White, Amos Kipkorir Langat

**Affiliations:** a Ecole de santé publique, Université Libre de Bruxelles, Bruxelles, Belgium; b Department of Research, Medical Research Circle (MedReC), Goma, DR Congo; c Faculty of Medicine, Official University of Bukavu, Bukavu, DR Congo; d Department of Medicine, Karachi Medical and Dental College, Karachi, Pakistan; e Department of Medicine, Dow university of Health and Sciences, Karachi, Pakistan; f Department of Research and Development, Shing Huei Group, Taipei, Taiwan; g Département de nutrition et diététique, Institut Supérieur des techniques médicales, Kinshasa, DR Congo; h International Veterinary Vaccinology Network, The Roslin Institute University of Edinburgh, Edinburgh, United Kingdom; i Department of Research, The Operating Room Global, Kigali, Rwanda; j Pan African University for Basic Sciences Technology and Innovation, Nairobi, Kenya.

**Keywords:** diphtheria, tetanus, tetanus vaccine, women

## Abstract

Tetanus poses a significant health threat, particularly in resource-limited countries. Its prevention is essential at all levels of health, primarily through vaccination. This study helps identify the factors responsible for low tetanus vaccination coverage among women of reproductive age. This cross-sectional study was conducted in 8 randomly selected health areas within an urban health zone and involved 358 women of reproductive age. This study was carried out using a well-structured questionnaire. Among the participating women, 86.3% had received both doses of Td2+ vaccine. The main factors explaining the observed low coverage were the lack of awareness among women by health workers regarding the importance of vaccination, insufficient knowledge of the vaccination schedule (*P* < .014), the women’s occupation (*P* < .001), nonattendance at antenatal consultations (*P* < .001), and noncompliance with appointments set by vaccination workers (*P* < .001). To improve tetanus vaccination coverage in an urban health zone, it is necessary to increase women’s awareness of the importance of vaccination, ensure compliance with the vaccination schedule, and ensure that appointments set by vaccination agents are kept.

**Key points**

- Cross-sectional study in an urban health zone in Bukavu, DR Congo. Comprised 358 women of reproductive age (15–45 years). 86.3% received both doses of the tetanus toxoid (Td2+) vaccine.
- The most salient barriers to universal coverage include inadequate health education, low vaccination schedule awareness, and failed prenatal consultations.
- Noncompliance with appointments and the absence of reminders decreased coverage.
- Recommendations include greater awareness, improved appointment keeping, and the integration of vaccination into antenatal care.

## 1. Introduction

Tetanus is a major public health problem worldwide. Although tetanus has become rare in developed countries, it remains a significant threat in resource-limited settings where vaccination coverage is inadequate.^[1]^ Most reported tetanus cases occur as a result of unsanitary conditions during childbirth, abortions, or postnatal care, and primarily affect unvaccinated mothers or their newborns.^[2]^ Tetanus and diphtheria are both very serious and potentially fatal bacterial infections; vaccination with the Td2+ (tetanus-diphtheria) vaccine can effectively prevent both infections.^[3]^ However, gaps in vaccination coverage remain a persistent concern in many regions, particularly in health zones with socioeconomic and logistical challenges. In the Democratic Republic of Congo (DR Congo), Td2+ vaccine coverage was very low with variations across different provinces.^[4,5]^ This study aims to identify and analyze the factors influencing insufficient vaccination coverage against tetanus and diphtheria among women of reproductive age. Identifying and understanding these factors is crucial in order to not only improve vaccination strategies and ensure adequate protection for this vulnerable population, which is particularly important due to its implications on maternal and child health, but also to propose appropriate recommendations to strengthen vaccination programs and improve access and acceptability of vaccines in this specific region.

## 2. Methodology

### 2.1. Study setting and design

This was a cross-sectional, community-based survey conducted between August and October 2024 in the Kadutu Urban Health Zone, in Bukavu, capital of South Kivu Province, in the eastern DR Congo. This region is characterized by high population density and the notable presence of various healthcare facilities.

### 2.2. Study population

This survey focused on the female population, specifically targeting women aged 15 to 45 years residing in the Kadutu Urban Health Zone. This age group was selected due to the inclusion of women of reproductive age, who are often at increased risk of not receiving tetanus toxoid (Td2+) vaccination, especially during the childbearing periods.

### 2.3. Inclusion and exclusion criteria

#### 2.3.1. Inclusion criteria

Women aged 15 to 45 years, residing in the Kadutu Urban Health Zone during our study period and who provided informed consent were included in this study.

#### 2.3.2. Exclusion criteria

Women under the age of 15 and those with a mental or cognitive disorder preventing their participation were excluded from this study. Those not residing in the Kadutu Health Zone or who did not provide informed consent were also not included.

#### 2.3.3. Sample size

To determine our sample size, we used a two-stage representative random sampling method based on the Schwartz formula:


$$\begin{array}{l} N=z{}^{2}\alpha x\left(\;p\times q\right)/I^{2} \end{array}$$

where N = sample size, *zα* = reduced deviation corresponding to the accepted risk *α*, 1.96 or 2, *P* = proportion of women who received at least 2 doses of tetanus vaccine in the Kadutu Health Zone = 61.3%, *q* = complement of *P* = 1 ‐ *p*, *I* = precision = 5%. Thus, N = 22 × 0.316 × 0.684/(0.05)² = 379.5, rounded to 380.

### 2.4. Data collection tools and procedures

Information was collected using a structured questionnaire, developed based on a literature review and validated by the research team. The questionnaire included the following sections: sociodemographic characteristics and factors associated with non-vaccination. The surveys were conducted face-to-face by trained interviewers speaking French and the local language.

### 2.5. Data analysis

Data were entered and processed using Microsoft Office Excel 2020 and Stata 15.1 software. Descriptive statistics (frequencies, percentages) were used to summarize the data. Variables with a *P* < .2 were included in a multivariate logistic regression model to identify independent factors associated with insufficient vaccination coverage.

### 2.6. Ethical considerations

Ethical approval for this study (Ethics Committee Ref/ERB/005/2025) was provided by the Ethics Committee of the Medical Research Circle (MedReC) in the Democratic Republic of Congo. The study protocol incorporated the segregation of patient identification data using confidentiality codes to ensure anonymity and protect the privacy of participants. This research was conducted in accordance with the ethical standards established in the 1964 Declaration of Helsinki and its later amendments, or comparable standards.

## 3. Results

In this study, based on the sociodemographic distribution (Table 1), the age group that is most represented is that between 15 and 25 years with 52.5% and 80.1% of these women are married and among them, 67.3% have a secondary education level. In our study population 15% of women have no knowledge about the disease against which they are vaccinated and the majority do not know the vaccination schedule in Td2+ (72.9%). Of these participants, 57.2% had attended <3 prenatal consultations and 13.6% have never received a tetanus vaccine.

**Table 1:** Distribution of sociodemographic characteristics of participants.

Features	Effective
**Age group**	
15–25	188 (52.5)
26–35	154 (43)
36–45	16 (4.4)
**Marital status**	
Divorcee	50 (13.9)
Bride	287 (80.1)
Widow	21 (5.8)
**Level of study**	
None	8 (2)
Primary	41 (11.4)
Secondary	241 (67.3)
Superior	68 (18.9)
**Occupation**	
Unemployed	35 (9.7)
Shopkeeper	95 (26.5)
Official	26 (7.2)
Housewife	202 (56.4)
**Knowledge about tetanus**	
Heard about	
Never heard of it	304 (84.9)
Knowledge of the tetanus vaccination schedule	54 (15)
No	261 (72.9)
Yes	97 (27)
**CPN followed**	
None	68 (18.9)
<3 CPN	205 (57.2)
>3 CPN	85 (23.7)

In Table 2, we note that in our study population, 78.7% of women received no explanation regarding tetanus vaccination and almost half of the women surveyed (50.8%) did not receive an appointment from the vaccination agent to receive the next dose of Td2+. And for 176 women who received the appointment, we find that 79.5% had kept the appointment.

**Table 2:** Distribution of women surveyed according to whether or not the vaccination officer provided explanations about tetanus vaccination.

Features	Effective
**Explanation of vaccination by agent (n = 358**)	
No	282 (78.7)
Yes	76 (21.2)
**Appointment given by the vaccination agent (n = 358**)	
No	182 (50.8)
Yes	176 (49)
**Meeting the appointment (n = 176**)	
No	36 (20.4)
Yes	140 (79.5)

Table 3 shows that the age group of 15 to 25 years was the most represented and therefore is the one that most received the vaccine (86.2%). In the other age groups, the proportion having received the vaccine remains always majority although we do not have a link between age and the receipt of the tetanus vaccine. In relation to the profession, the most represented women were housewives and among them 96% were able to receive the vaccine and we found a statistically significant correlation link between the profession and the receipt of the vaccine with a *P* < .001. We also find that 23.2% did not keep the appointment and therefore they did not receive the vaccine. And therefore, a link between the respect of the appointment given by the agent and the receipt of the tetanus vaccine could be found with a statistically significant value *P* < .001.

**Table 3:** Factors associated with non-vaccination.

Features	Td2+ vaccine received		
	No	Yes	
	Effective	Effective	
**Age**			*P* = .250
15–25 (n = 189)	26 (13.7)	163 (86.2)	
26–35 (n = 153)	29 (18.9)	124 (81)	
36–45 (n = 16)	0 (0)	16 (100)	
**Educational level**			*P* = .170
None (n = 8)	0 (0)	8 (2.2)	
Primary (n = 41)	3 (7.3)	38 (92.6)	
Secondary (n = 241)	33 (13.6)	208 (86.3)	
Superior (n = 68)	13 (19.1)	55 (80.8)	
Occupation			*P* < .001
Unemployed (n = 35)	6 (17.1)	29 (82.8)	
Shopkeeper (n = 95)	31 (32.6)	64 (67.3)	
Civil servant (n = 26)	4 (15.3)	22 (84.6)	
Housewife (n = 202)	8 (3.9)	194 (96)	
CPN monitoring			*P* < .001
No (n = 68)	30 (44.1)	38 (55.8)	
Yes (n = 290)	19 (6.5)	271 (93.4)	
CVA knowledge			*P* = .014
No (n = 261)	45 (17.2)	216 (82.7)	
Yes (n = 97)	4 (4.1)	93 (95.8)	
Appointment kept			*P* < .001
No (n = 189)	44 (23.2)	145 (76.7)	
Yes (n = 169)	5 (2.9)	164 (97)	
Marital status			*P* = .336
Divorced (n = 50)	4 (8)	46 (92)	
Married (n = 287)	42 (14.6)	245 (85.3)	
Widow (n = 21)	3 (14.2)	18 (85.7)	

44.1% of the women surveyed did not attend a prenatal consultation and therefore did not receive the vaccine either. There is a correlation between attendance at prenatal consultations and receipt of the vaccine. However, there is no link between marital status and receipt of the tetanus vaccine given the statistically less significant threshold with a *P* = .33.

## 4. Discussion

This cross-sectional study assessed Td2+ vaccination coverage among women of reproductive age in an urban health zone of Bukavu, Democratic Republic of Congo. Although 86.3% of participants reported receiving the vaccine, considerable gaps in knowledge and health engagement were observed. About 72.9% were unaware of the recommended vaccination schedule, and 15% lacked understanding of the disease being prevented. Vaccination status showed significant associations with occupation, antenatal care attendance, and adherence to vaccination appointments (*P* < .001 for each), highlighting the impact of both individual and health system-level factors on immunization coverage.^[1,6]^

These findings have important implications for improving maternal and neonatal tetanus prevention. While coverage appears relatively high, inadequate awareness among women and weak communication from healthcare providers remain barriers. The significant link between antenatal visits and vaccination status aligns with existing evidence that maternal health services improve immunization uptake.^[7]^ A multicountry analysis of sub-Saharan Africa showed that 4 or more antenatal visits, higher maternal education, and institutional deliveries substantially increased tetanus vaccination rates.^[8]^ Moreover, communication by trusted healthcare workers plays a crucial role. Positive provider attitudes and patient–provider dialogue have been shown to boost maternal acceptance of vaccines during pregnancy.^[4]^ Enhancing training for health workers in education and interpersonal communication could address these gaps.^[5,9]^

This study’s results are consistent with previous research emphasizing the influence of maternal education and socioeconomic status. In Kenya, children of mothers with formal education were significantly more likely to be vaccinated, highlighting the importance of maternal knowledge in health decision-making.^[10]^ Similarly, a study across 45 low- and middle-income countries found strong links between maternal education, household wealth, and child immunization.^[6]^ Wealth-related inequalities also persist, with children in higher-income households more likely to receive timely vaccinations.^[11]^ These patterns suggest that health promotion strategies should address structural inequalities, not just behavioral gaps.

More recently, studies from Nigeria and Zambia have demonstrated that integrating tetanus vaccination with routine maternal care, particularly during antenatal consultation visits, significantly boosts uptake.^[12,13]^ A study in Ethiopia further emphasized the role of male partner support and maternal autonomy in health decisions.^[14,15]^ These findings highlight the multifactorial nature of vaccine uptake and the need for community-level engagement in addition to facility-based interventions.

### 4.1. Limitations

This study has limitations. Its cross-sectional design precludes causal inference. Self-reported data may introduce recall bias, particularly regarding vaccination status and antenatal visits. The exclusion of incomplete questionnaires, though necessary for data integrity, could result in selection bias. Moreover, findings from this urban setting may not generalize to rural populations, where access to healthcare and information may differ substantially.

Future studies should explore the sociocultural and behavioral drivers of vaccine hesitancy among women, including beliefs about safety and perceived relevance. Qualitative research could help uncover personal and community-level barriers. Longitudinal designs could evaluate how educational interventions influence vaccine uptake over time. Research on mobile health tools, outreach programs, and integration with antenatal consultation services may further inform national immunization strategies. Importantly, studies targeting rural and marginalized communities are vital for promoting equitable vaccine access.

## 5. Conclusion and recommendations

Tetanus continues to be an avoidable yet persistent health condition in poor settings such as the DR Congo, hugely impacting women of reproductive age and their offspring. This study showed a relatively high vaccination coverage rate (86.3%) among citizens within Kadutu’s urban health zone, yet it significantly disclosed several factors responsible for the non-vaccination. These factors include low awareness campaigns by healthcare providers, women’s poor knowledge of the vaccination schedule, discrepancies in occupational levels, poor attendance at prenatal clinics, and poor follow-up of the vaccination sessions.

One of the most striking results was the absence of communication between the healthcare workers and the women they serve. Over 78% of the participants reported not being informed about the tetanus vaccine. Furthermore, nearly half of the women did not receive follow-up Td2+ appointments, and among those who did, compliance was patchy and based on their understanding and commitment. These gaps in service delivery undermine public health efforts considerably, even with the availability of vaccines.

The occupational history of the respondents also had a significant influence. Women identified as housewives had the highest rate of vaccine compliance, likely due to greater flexibility and availability to attend health services. Women with demanding occupations like shopkeepers had significantly lower levels of vaccine series completion. This suggests the need to design vaccination programs in a manner responsive to the working realities of women, such as flexible scheduling and community-based outreach.

Prenatal visits became an important determinant in this context. Women who did not visit antenatal care had a significantly higher risk of missing vaccinations. This highlights the dual importance of strengthening maternal healthcare services and integrating vaccination promotion into routine prenatal visits.

According to these results, the following are the recommendations made:

1. *Improve health education and communication:* It is essential that health workers place utmost importance on giving information on vaccination during consultations, emphasizing its significance, particularly among first-time mothers and young women.
2. *Put systematic follow-up processes in place:* Health facilities can put in place effective appointment-tracking systems and utilize SMS reminders or community health workers to bring women to their appointments and ensure they return.
3. *Add immunization services to women’s routines:* Additional hours or outreach clinics can be scheduled for working women, including those in the informal sector.
4. *Add immunization to antenatal services:* Tetanus immunization has to be done as part of antenatal services, which has to be done with compulsory counseling at every visit.
5. *Encourage and educate health workers:* Health workers can be motivated to promote the needed messages and follow up on missed opportunities for vaccination with incentive rewards and training done regularly.
6. *Organize the community:* Health educators and community heads can assist greatly in establishing confidence within the community and in accepting the vaccine.

By interventions targeting these issues of multiple causal factors, maternal vaccination coverage can be greatly improved, newborns safeguarded, and the ultimate goal of maternal and neonatal tetanus elimination furthered. The present work offers a critical body of evidence from which to obtain context-specific and data-informed public health interventions in Congolese urban and similar environments.

Tetanus vaccination coverage among reproductive-age women in the urban health zone of Bukavu was high, yet major gaps in awareness and system-level communication persist. These challenges, along with socioeconomic disparities, hinder full immunization. Addressing them will require integrated approaches that combine health provider training, improved patient education, and equitable service delivery. Strengthening antenatal care linkages and targeting health promotion efforts to underserved groups could enhance maternal and neonatal protection against tetanus.

## Acknowledgments

The authors would like to thank the direction of research and the collaboration with the Medical Research Circle (MedReC) of Democratic Republic of the Congo for the realization of this present paper.

## Author contributions

**Conceptualization:** Eric Buchangende Ndagano, Rodrigue Fikiri Bavurhe, Christian Bisimwa Wabene, Prudent Mituga Ngabe, Aymar Akilimali.

**Data curation:** Eric Buchangende Ndagano, Rodrigue Fikiri Bavurhe, Christian Bisimwa Wabene, Prudent Mituga Ngabe, Aymar Akilimali.

**Formal analysis:** Eric Buchangende Ndagano, Rodrigue Fikiri Bavurhe, Aymar Akilimali.

**Funding acquisition:** Eric Buchangende Ndagano, Rodrigue Fikiri Bavurhe, Aymar Akilimali, Amos Kipkorir La.

**Investigation:** Eric Buchangende Ndagano, Rodrigue Fikiri Bavurhe, Aymar Akilimali.

**Methodology:** Eric Buchangende Ndagano, Rodrigue Fikiri Bavurhe, Zehra Sadia, Rabbia Munsab, Aymar Akilimali.

**Project administration:** Eric Buchangende Ndagano, Rodrigue Fikiri Bavurhe, Rabbia Munsab, Aymar Akilimali, Amos Kipkorir Langat.

**Resources:** Eric Buchangende Ndagano, Rodrigue Fikiri Bavurhe, Aymar Akilimali, Amos Kipkorir Langat.

**Software:** Eric Buchangende Ndagano, Rodrigue Fikiri Bavurhe, Aymar Akilimali.

**Supervision:** Eric Buchangende Ndagano, Rodrigue Fikiri Bavurhe, Rabbia Munsab, Calvin R. Wei, Aymar Akilimali, Jonathan White, Amos Kipkorir Langat.

**Validation:** Eric Buchangende Ndagano, Rodrigue Fikiri Bavurhe, Zehra Sadia, Calvin R. Wei, Aymar Akilimali, Jonathan White, Amos Kipkorir Langat.

**Visualization:** Eric Buchangende Ndagano, Rodrigue Fikiri Bavurhe, Rabbia Munsab, Calvin R. Wei, Aymar Akilimali, Jonathan White.

**Writing – original draft:** Eric Buchangende Ndagano, Rodrigue Fikiri Bavurhe, Zehra Sadia, Rabbia Munsab, Calvin R. Wei, Aymar Akilimali.

**Writing – review & editing:** Eric Buchangende Ndagano, Rodrigue Fikiri Bavurhe, Zehra Sadia, Rabbia Munsab, Calvin R. Wei, Aymar Akilimali.
